# Enteral feeding practices and guideline adherence for very low birth weight infants in Chinese NICUs: a multicentre cross-sectional survey

**DOI:** 10.3389/fnut.2026.1868242

**Published:** 2026-07-08

**Authors:** Yu Wu, Yuxia Zhang, Xiaoqin Liu, Xin Wang, Mimi Li, Tiantian Yan, Zhongxia Zhu, Shuhuan Song, Xiaoman Yu, Ying Shen, Hong Jiang, Aihong Liu, Minmin Li

**Affiliations:** 1 Second Ward, Department of Obstetrics of Olympic Branch, Shandong Provincial Hospital Affiliated to Shandong First Medical University, Jinan, Shandong, China; 2 Department of Pediatrics, Qilu Hospital of Shandong University, Jinan, Shandong, China; 3 Neonatal Intensive Care Unit, Shandong Provincial Hospital Affiliated to Shandong First Medical University, Jinan, Shandong, China; 4 Department of Pediatrics, Qingdao Rehabilitation University Hospital (Qingdao Municipal Hospital), Qingdao, Shandong, China; 5 Neonatal Intensive Care Unit, Zaozhuang Municipal Hospital, Zaozhuang, Shandong, China; 6 Maternity and Child Health Care of Zaozhuang, Zaozhuang, Shandong, China; 7 Neonatal Ward of the Third Department of Pediatrics, The Affiliated Taian City Centeral Hospital of Qingdao University, Tai’an, Shandong, China; 8 Department of Neonatal, Liaocheng Second People’s Hospital, Liaocheng, Shandong, China; 9 Neonatal Intensive Care Unit, The Affiliated Hospital of Qingdao University, Qingdao, Shandong, China; 10 Department of Pediatrics, Affiliated Hospital of Shandong Second Medical University, Weifang, Shandong, China; 11 Neonatal Intensive Care Unit, Yantai Yuhuangding Hospital, Yantai, Shandong, China; 12 Neonatal Intensive Care Unit, The Second Qilu Hospital of Shandong University, Jinan, Shandong, China; 13 Neonatal Intensive Care Unit of Olympic Branch, Shandong Provincial Hospital Affiliated to Shandong First Medical University, Jinan, Shandong, China

**Keywords:** clinical practices, enteral feeding, feeding strategies, NICU, VLBW infant

## Abstract

**Objectives:**

This study aimed to investigate the current status of enteral feeding for VLBW infants in NICUs, and to assess compliance with guidelines and evidence.

**Methods:**

Online survey on enteral feeding practices for VLBW infants was conducted from August to November 2024. The Quick Response Code and link of this survey were distributed to partner institutions of the Shandong Nursing Association. The results were compared with recommendations from the evidence and guidelines on enteral nutrition for VLBW infants.

**Results:**

47 NICUs were invited to participate in this survey. Among these, 32 NICUs (68.1%) had established the standardized feeding protocol specifically designed for VLBW infants. All institutions prioritize breast milk as the preferred choice, but 53.2% of NICUs have breast milk or donated breast milk available as the source of milk for initiating enteral feeding. 39 NICUs (83.0%) initiate enteral feeding within 24 h. 41 NICUs (87.3%) administered enteral feeding every 2–3 h, with a daily volume increase of 13 (2, 15) ml. NICUs defined full enteral feeding based on either caloric or volume targets, established in accordance with guidelines and local experience. Specifically, 27 NICUs (57.4%) defined it as a mean intake of (123.4 ± 21.8) kcal/kg/day, and 37 NICUs (78.7%) defined it as (138.4 ± 35.7) ml/kg/day. Initial enteral feeding volumes and the time required to reach full enteral feeding were managed differently based on birth weight. Human milk fortification was initiated when the infant’s intake reached (74.9 ± 33.7) ml/kg/day, but only 20 NICUs (42.6%) adopted it as a routine nutritional support measure. Approximately 30% of institutions routinely measured gastric residual volume. Depending on the volume, gastric residuals were either re-fed to the infant or discarded.

**Conclusion:**

While the clinical practices were aligned with evidence and guidelines for VLBW infants, divergence remained in areas such as the use and timing of human milk fortification. This study not only provides regional data but also analyzes implementation gaps between evidence and clinical practice. By analyzing these discrepancies, this study identified deficiencies in nutritional management within NICUs and provides a basis for continuous quality improvement in the care of preterm infants.

## 1 Introduction

Preterm birth is the most significant cause of death during the neonatal period, resulting in approximately one million neonatal deaths annually. Compared with late preterm infants and low birth weight infants, very low birth weight (VLBW) infants and very premature infants are at significantly higher risk of complications such as infections (1). Optimizing early nutrition for VLBW infants could promote their long-term health outcomes and reduce the incidence of complications by improving their growth and neurodevelopment (2). Preterm or low birth weight infants, particularly those who are very preterm or very low birth weight, have few nutrient reserves at birth. Physiological and metabolic stresses and adaptations after birth further increase their nutritional requirements (3). Failure to achieve an optimal rate of postnatal growth similar to that of uncompromised fetuses, or to meet nutritional requirements aligned with the growth trajectory that occurred in utero at the equivalent gestational age, may lead to growth restriction (4) and even developmental disorders during childhood and adolescence, resulting in long-term neurodevelopmental and subsequent growth outcomes (5). Therefore, implementing proactive and structured feeding strategies is essential to address their elevated nutritional requirements and potential growth limitations. Substantial heterogeneity exists in enteral feeding practices for VLBW infants across countries and institutions. Nutritional management and enteral feeding for preterm infants vary by weight. Infants weighing approximately 1,500 g or more are typically fed orally or via an intragastric feeding tube from birth, preferably with human milk, which is similar to the feeding strategy for full-term infants. In contrast, extremely low birth weight (ELBW) infants who weigh less than 1,000 g initially require parenteral nutrition, with trophic feeds introduced within the first week, followed by a gradual transition to enteral nutrition (6). However, regarding nutritional management for preterm infants weighing between 1,000 g and 1,500 g, the policy and practice of high-income countries has tended to favour the conservative approach to advancing enteral feeds. Whereas in low- and middle-income countries, constraints in NICUs resources often lead to more pragmatic approaches that tend to favour the early introduction and advancement of enteral feeds (7). For example, enteral feeding for VLBW infants in Shanghai is initiated within 30 h after birth, with full feeding achieved in approximately 30 days. In contrast, data from Guangdong indicate later initiation and a longer duration to reach full enteral feeding (8). The chosen feeding strategy should always prioritize preterm infant safety to minimize the risk of severe and potentially fatal complications such as necrotising enterocolitis (NEC) (9). However, evidence shows that delaying or slowly advancing enteral feeding can lead to intestinal mucosal atrophy and bacterial translocation, increasing the incidence of subsequent complications, including late-onset sepsis (LOS) (10, 11). Each additional one-week delay in achieving full enteral feeding was associated with a 16% higher relative risk of LOS (12). Compared with a conservative approach, earlier and faster feeding advancement did not increase the risk of NEC but was associated with a lower incidence of LOS (13). Therefore, it is important to develop clear, practical guidelines that can effectively translate into clinical practice.

A multi-disciplinary working group at McMaster University, Canada, developed and published the Guidelines for Feeding Very Low Birth Weight Infants. These guidelines were based on a structured literature search and rigorous evidence evaluation (14). Although published some time ago, this guideline remains a widely recognized and authoritative reference on enteral nutrition for VLBW infants by scholars both domestically and internationally (15, 16). High-quality, evidence-based clinical practice guidelines for enteral nutrition in preterm infants have also been established by international societies, including the Turkish Neonatal Society in 2018 and the European Society of Pediatric Gastroenterology, Hepatology and Nutrition in 2022 (15, 17). Meanwhile, the Chinese Medical Doctor Association published evidence and expert consensus that the main basis for the management of enteral nutrition for premature infants in China, including Shandong Province (18, 19). However, these evidences were developed for the broader population of preterm infants. The unique clinical characteristics of VLBW infants may render some recommendations and interventions unsuitable for direct application. Consequently, in the absence of the updated, comprehensive, and targeted guideline recommendations for enteral nutrition of VLBW infants, it is necessary to synthesize recommendations from the Canadian guidelines (2015), Turkish guidelines (2018), European guidelines (2020), and Chinese guidelines (2024), while also referencing other evidence to address discrepancies in clinical practice (20, 21).

The clinical practice and management of enteral feeding for VLBW infants in China, including Shandong Province, are primarily based on guidelines and clinical experience. To identify and analyze deficiencies and determinants in clinical practice, thereby enhancing guideline adherence and the scientific rigor of clinical decision-making, it is essential to analyze the variation in clinical practice across China and compare these implementation patterns with evidence-based guidelines. Accordingly, we conducted a multicenter survey in Shandong Province to investigate the current status and adherence to guidelines of enteral feeding practices for VLBW infants in NICUs. This study aimed to provide a reference for meeting their nutritional requirements and advancing enteral nutrition.

## 2 Materials and methods

### 2.1 Study design

The Neonatal Nursing Committee of the Shandong Nursing Association conducted a province-wide, cross-sectional, and descriptive survey. The survey aimed to investigate the current status of enteral feeding practice of NICUs across Shandong Province and was administered between August and November 2024.

### 2.2 Measures

A closed-ended questionnaire was developed based on a comprehensive review of the evidence and guidelines on enteral nutrition for VLBW infants. The questionnaire consisted of two parts. 10 neonatologists and neonatal nurses were selected to assess the clinical practice using this questionnaire for the pilot test. All participants reported no misunderstanding of items. A panel of 8 experts in enteral feeding management for VLBW infants assessed the questionnaire’s relevance, accuracy, and comprehensibility. The results show that S-CVI/Ave was 0.917, and the I-CVI ranged from 0.875 to 1.000.

Part One of the questionnaire comprises items on institutional characteristics without patient-level data, including the hospital’s name, category, NICU level, number of neonatologists and neonatal nurses, standardized feeding protocol or workflow, and training sessions. Part Two focuses on enteral feeding practices for VLBW infants, including timing of initiation, choice of initial milk, definition of full enteral feeding, and use of human milk fortification (HMF), and so on. Preliminary surveys conducted by research team members indicate that each questionnaire takes approximately 3 to 10 min to complete.

### 2.3 Data collection

The questionnaire was developed and distributed electronically using a survey platform to facilitate data collection and management. The Quick Response Code (QR code) and link were distributed to partner institutions of the Neonatal Nursing Committee of the Shandong Nursing Association. After data collection, data cleansing and logical verification were performed to ensure data integrity and validity. As part of the quality control procedure, questionnaires completed in less than 2 min or with missing items were considered invalid. Institutions that submitted incomplete or invalid questionnaires were requested to resubmit valid responses from their NICUs. The questionnaires were completed by the head nurse or designated representative at each institution. To ensure representativeness and consistency, each organization may submit only one completed questionnaire. If any institution submitted more than two questionnaires or had incomplete responses, it was requested to review and ultimately submit a unique valid questionnaire. A total of 50 NICUs were invited, with 47 returning valid questionnaires, yielding a response rate of 94% (2 NICUs declined, and 1 NICU was excluded due to missing data).

### 2.4 Participants

This survey collected data from 47 medical institutions, including 39 Level III NICUs, 8 Level II NICUs, 31 general hospitals, and 16 specialized hospitals. This study employed a descriptive analysis to summarize differences across different hospital categories and NICU levels regarding the timing of enteral feeding, daily milk volume increments, and the timing of HMF use.

### 2.5 Ethics approval

This study was conducted in accordance with the Declaration of Helsinki. The Ethics Committee of Shandong Provincial Hospital Affiliated to Shandong First Medical University was consulted before data collection. The committee indicated that formal ethical review was not necessary, given that the study was an anonymous, institutional-level survey of NICU policies that did not involve any personal or private data on patients or healthcare professionals. Nonetheless, all participating NICUs obtained institutional approval through their respective administrative departments. The NICU head nurses or designated representatives were informed of the study’s purpose and design. Completion and submission of the questionnaire was considered implied consent.

### 2.6 Statistical analyses

R 4.5.2 and Microsoft Excel 2023 were used for data collation and statistical analysis. Depending on the data type, quantitative data were presented as mean ± standard deviation (SD) or medium (interquartile range) based on whether they were normally distributed. Categorical data were presented as frequencies (percentages).

## 3 Results

### 3.1 Study population

This survey included 47 NICUs across medical institutions. Among these, 32 NICUs (68.1%) had established a standardized feeding protocol or workflow specifically designed for VLBW infants (see Table 1).

**Table 1:** Demographic characteristics of institutions.

Items		Level of NICU		Category of hospital		Total
		Level III units	Level II units	General hospital	Specialized hospital	
Bed number of NICU		38.7 ± 19.9	28.0 ± 34.1	33.8 ± 17.9	42.8 ± 30.1	36.9 ± 22.9
Number of neonatologists		12.8 ± 6.7	8.9 ± 5.6	11.4 ± 5.3	13.6 ± 8.7	12.1 ± 6.7
Number of neonatal nurses		33.9 ± 16.9	16.9 ± 9.4	30.9 ± 16.5	31.1 ± 18.6	31.0 ± 17.1
A standardized feeding protocol or workflow specifically designed for VLBW infants	Yes	30 (76.9)	2 (25.0)	23 (74.2)	9 (56.3)	32 (68.1)
	No	9 (23.1)	6 (75.0)	8 (5.8)	7 (43.7)	15 (31.9)
Number of training sessions for standardized feeding protocol or workflow	0	4 (10.3)	2 (25.0)	3 (9.7)	3 (18.8)	6 (12.8)
	1–2	28 (71.8)	5 (62.5)	22 (71.0)	11 (68.7)	33 (70.2)
	≥3	7 (17.9)	1 (12.5)	6 (19.3)	2 (12.5)	8 (17.0)

### 3.2 Enteral feeding practices for VLBW infants

The survey revealed that enteral feeding practices for VLBW infants varied across different hospital categories and NICU levels within Shandong Province (see Table 2).

**Table 2:** Practice status of enteral feeding practices in preterm infants with VLBW.

Item		Level of NICU		Category of hospital		Total
		Level III units	Level II units	General hospital	Specialized hospital	
Initiation of enteral feeding	<24 h	33 (84.6)	6 (75.0)	27 (87.1)	12 (75.0)	39 (83.0)
	>24 h	6 (15.4)	2 (25.0)	4 (12.9)	4 (25.0)	8 (17.0)
Type of milk for initiating enteral feeding	Breast milk or donor human milk	19 (48.7)	6 (75.0)	15 (48.4)	10 (62.5)	25 (53.2)
	Preterm formula	14 (35.9)	2 (25.0)	10 (32.3)	6 (37.5)	16 (34.0)
	Protein hydrolyzed formulas	6 (15.4)	0 (0.0)	6 (19.3)	0 (0.0)	6 (12.8)
Volume of milk for initiating enteral feeding	<1,000 g	–	–	–	–	10.3 ± 5.3
	1,000 ~ 1,500 g	–	–	–	–	15.9 ± 8.4
Frequency of enteral feeding	continuous feeding	1 (2.6)	0 (0.0)	0 (0.0)	1 (6.25)	1 (2.1)
	Two-hourly	15 (38.4)	5 (62.5)	12 (38.7)	8 (50.0)	20 (42.6)
	Three-hourly	20 (51.3)	1 (12.5)	15 (48.4)	6 (37.5)	21 (44.7)
	Six-hourly	3 (7.7)	2 (25.0)	4 (12.9)	1 (6.25)	5 (10.6)
Daily increase in feeding volume		–	–	–	–	13 (2.15)
Definition of full enteral feeding	kcal/kg/day	–	–	–	–	123.4 ± 21.8
	mL/kg/day	–	–	–	-	138.4 ± 35.7
Timing of full enteral feeding	<1,000 g	–	–	–	–	18.9 ± 9.0
	1,000 ~ 1,500 g	–	–	–	–	12.6 ± 5.3
Using human milk fortification	Always	18 (46.2)	2 (25.0)	14 (45.2)	6 (37.5)	20 (42.6)
	Often	15 (38.4)	3 (37.5)	11 (35.5)	7 (43.7)	18 (38.3)
	Occasionally	3 (7.7)	0 (0.0)	3 (9.7)	2 (12.5)	5 (10.6)
	Sometimes	3 (7.7)	2 (25.0)	2 (6.4)	1 (6.3)	3 (6.4)
	Never	0 (0.0)	1 (12.5)	1 (3.2)	0 (0.0)	1 (2.1)
Timing of using human milk fortification (*n* = 43)		–	–	–	–	74.9 ± 33.7
Routine measurement of gastric residual volumes	Yes	12 (30.8)	2 (25.0)	8 (25.8)	6 (37.5)	14 (29.8)
	No	27 (69.2)	6 (75.0)	23 (74.2)	10 (62.5)	33 (70.2)
Management of Residuals that is GRV is <50% of the previous feeding volume	Re-feeding the aspirate and skipping the current feed	2 (5.1)	1 (12.5)	1 (3.2)	2 (12.5)	3 (6.4)
	Re-feeding the aspirate and administering the current feed	9 (23.1)	2 (25.0)	5 (16.1)	6 (37.5)	11 (23.4)
	Re-feeding the aspirate and giving the feed up to the total feeding volume	25 (64.1)	5 (62.5)	22 (71.0)	8 (50.0)	30 (63.8)
	Discarding the aspirate and administering the current feed	3 (7.7)	0 (0.0)	3 (9.7)	0 (0.0)	3 (6.4)
Management of Residuals that is GRV is >5 mL/kg and >50% of the previous feeding volume	Re-feeding the aspirate up to 50% of the total feeding volume and skipping the current feed	17 (43.6)	2 (25.0)	12 (38.7)	7 (43.7)	19 (40.4)
	Re-feeding the aspirate and skipping the current feed	14 (35.9)	1 (12.5)	14 (45.2)	1 (6.3)	15 (31.9)
	Discarding the aspirate and skipping the current feed	6 (15.4)	4 (50.0)	4 (12.9)	6 (37.5)	10 (21.3)
	Discarding the aspirate and administering the current feeding	2 (5.1)	1 (12.5)	1 (3.2)	2 (12.5)	3 (6.4)

#### 3.2.1 Initiating enteral feeding

All institutions prioritize breast milk as the preferred choice, but only 53.2% of NICUs have breast milk or donated breast milk available as the source of milk for initiating enteral feeding for VLBW infants. For infants weighing <1,000 g, the volume of milk for initiating enteral feeding is (10.3 ± 5.3) ml/kg/day. Infants weighing 1,000-1500 g receive (15.9 ± 8.4) ml/kg/day as the initial feeding volume with a daily volume increase of 13 (2, 15) ml/kg/day.

#### 3.2.2 Full enteral feeding

NICUs defined full enteral feeding based on either caloric or volume targets, established in accordance with guidelines and local experience. Specifically, 27 NICUs (57.4%) defined it as a mean intake of (123.4 ± 21.8) kcal/kg/day, and 37 NICUs (78.7%) defined it as (138.4 ± 35.7) ml/kg/day. The time to achieve this goal differed by birth weight, taking (18.9 ± 9.0) days for infants weighing <1,000 g, compared to (12.6 ± 5.3) days for those weighing 1,000-1500 g.

#### 3.2.3 Human milk fortification

The use of HMF was based on achieving an infant intake of (74.9 ± 33.7) ml/kg/day, but fewer than half of the institutions (42.6%) adopt it as a routine nutritional support measure.

#### 3.2.4 Management of residuals

To assess whether VLBW infants experience feeding intolerance, approximately 30% of institutions routinely measure gastric residual volume (GRV). When GRV is less than 50% of the feed volume, 63.8% of NICUs choose to re-feed the aspirate to the infant and giving feed up to the total feeding volume. When GRV exceeds 5 mL/kg and 50% of the feeding volume, 40.4% of institutions choose to re-feed the aspirate up to 50% of the total feeding volume and skip the current feed.

## 4 Discussion

This study conducted a comprehensive investigation of clinical practices regarding enteral feeding for VLBW infants in NICUs across Shandong Province, China. Comparative analysis revealed that most clinical practices were consistent with evidence and guidelines for VLBW infants. However, some differences remained between specific domestic practices and international evidence and guidelines.

### 4.1 Timing of initiation

83% of NICUs initiated enteral feeding within 24 h, while 17% initiated it more than 24 h after birth. This is consistent with the recommendations of Canadian guidelines and Chinese expert consensus documents, which all suggest that clinically stable infants should initiate enteral feeding within 24 h (14, 18). An increasing number of studies have found that early enteral feeding does not increase the risk of necrotizing enterocolitis or hypoglycaemia (22). Meanwhile, it shortens the duration of parenteral nutrition and reduces the incidence of LOS (23). Preclinical studies in mammals have also shown that intestinal mucosal reduction occurs within 48 h of NPO, and intestinal villus atrophy occurs within 72 h (24). Therefore, current recommendations tend to advocate early enteral feeding rather than delaying it. However, a recent multicenter cohort study in China has found that initiating enteral feeding within the first 24 h after birth was associated with an increased risk of NEC in specific subgroups, including infants delivered by cesarean section, those receiving blood transfusion, and those with a gestational age of less than 28 weeks (25). Importantly, this association was confined to these subgroups and not observed in the overall preterm population. In contrast, larger trials and cohort studies have consistently demonstrated that early initiation of enteral feeding does not increase the risk of NEC in most VLBW infants and may even reduce the incidence of LOS (22, 23). Delaying the introduction of progressive feeding was found to reduce the risk of feeding intolerance (26). Determining the optimal time for initiating enteral feeding in VLBW infants remains a clinical challenge. Thus, larger-scale, high-quality randomized controlled studies covering a wider range of special conditions, such as intraventricular hemorrhage (IVH) and patent ductus arteriosus (PDA), or predictive models for enteral feeding, need to be conducted through global collaboration to develop personalized feeding plans for infants in various clinical scenarios.

### 4.2 Rate and pattern of advancement

To reduce feeding intolerance in VLBW infants and lower the risk of developing severe intestinal diseases, most NICUs adopted a conservative feeding approach that gradually introduces enteral feeding, which is consistent with the findings of this study (27). The slow progression of enteral feeding may reduce the beneficial stimulation, delay the functional adaptation of the gastrointestinal tract, and disrupt the pattern of gut microbial diversity colonisation (28). Therefore, an increasing number of studies have found that conservative and slow progression of enteral feeding not only fails to reduce the incidence of NEC but also increases the risk of metabolic complications, slows growth and development, and prolongs hospital stays (29). Although current evidence supports the early initiation of full enteral feeding, its implementation remains highly challenging for NICU healthcare professionals, particularly in underdeveloped regions with shortages of NICU staff and monitoring equipment. Without precise medical equipment and experienced clinical judgment, it may not be possible to promptly identify abnormal clinical symptoms and signs in premature infants, leading to delays in diagnosis and treatment (30). Among the short-term to medium-term improvement measures, pragmatic strategies that do not require additional equipment are more immediately actionable and broadly applicable. These include standardized feeding protocols, staff training, structured feeding tolerance assessment, and avoidance of routine GRV checks. In the long run, emerging technologies such as abdominal near-infrared spectroscopy (NIRS) and complex feeding intolerance prediction scoring systems may hold theoretical promise for assisting routine monitoring of enteral feeding. However, these modalities are technically demanding, costly, and not yet widely accessible. Their potential role in improving the detection of nutrition-related complications and promoting the growth of VLBW infants will require further validation and cost-effectiveness evaluation before they can be recommended for routine clinical use.

### 4.3 Choice of initial milk

According to this survey, all institutions prioritize breast milk as the preferred choice, but only 53.2% of NICUs have breast milk or donated breast milk available as the source of milk for initiating enteral feeding for VLBW infants. All the guidelines and evidence indicate that the preferred type of milk for the first enteral feeding of VLBW infants is breast milk, followed by donated breast milk and preterm infant formula (14, 15, 17, 20, 21). There is no doubt that breast milk plays a crucial role in the growth and development of full-term, preterm, and very low birth weight infants (31). However, investigations have revealed that mothers separated from their infants often experience insufficient lactation due to a lack of adequate and high-quality breastfeeding support (32). They reported that they often received simple or repetitive health education on breastfeeding, but this has not been effective in promoting milk production and has instead increased the use of formula milk (33). Therefore, healthcare professionals should enhance their ability to support breastfeeding by offering systematic consultations and practical operational training to help these mothers achieve sufficient milk production, thereby enabling them to exclusively breastfeed their babies (34).

### 4.4 Use and timing of human milk fortification

Most guidelines recommend the use of HMF for VLBW infants (14, 15, 17, 20, 21). However, only 42.6% of NICUs adopted HMF as a routine nutritional support measure, a finding consistent with previous studies (35). Studies have found that HMF not only meets the nutritional requirements of VLBW infants but also reduces abnormal gut microbiota colonization, enhancing microbial diversity and uniformity (36, 37). Despite growing recognition of the benefits of HMF, its adoption remains limited, partly due to the high osmolarity of fortified milk (393 mOsm/kg versus 302 mOsm/kg for unfortified milk) and concerns about potential adverse effects such as NEC and sepsis (38). Additionally, the high cost of HMF restricts its use, particularly in low- and middle-income countries (38, 39). In such contexts, preterm formula powder fortification (PTF) may serve as a more affordable alternative to HMF, although it lacks the bioactive components of human milk. Adjustable fortification (AF) may offer greater individualization but requires more frequent monitoring and higher resource investment (40). Therefore, it may be more difficult to implement than simply procuring HMF or PTF in many NICUs.

### 4.5 GRV monitoring and management

The investigation found that 30.8% of NICUs routinely measured gastric residuals, a practice that deviates from guideline recommendations. Accumulating evidence suggests that discontinuing routine GRV measurement could shorten the time to reach full enteral feeding without increasing complications (41). For instance, the GRASS trial in infants with a gestational age 26–30 weeks found no difference in time to full feeds or major morbidities with routine GRV monitoring (42). Notably, routine GRV checks in clinically stable preterm infants have been associated with delayed feeding advancement and may increase the risk of NEC due to unnecessary feeding interruptions (43). However, abnormal residuals, particularly bloody or bilious aspirates, when interpreted together with other clinical signs, may raise suspicion of NEC but are not reliable as a standalone screening tool (43, 44). Thus, it is important to distinguish between targeted assessment of abnormal residuals within a broader clinical context and routine GRV checks in stable infants, which lack support from current evidence. Traditional monitoring methods may also cause mechanical injury to the gastric mucosa and disrupt the stability of the gut microbiota (44, 45). Point-of-care ultrasound (POCUS) may offer a non-invasive alternative for assessing gastric contents by measuring the cross-sectional area of the gastric antrum, and preliminary studies have shown its potential utility in both adult and pediatric nutritional and respiratory management (46–48). However, evidence in VLBW infants remains limited. Future research may explore whether POCUS-guided GRV monitoring could help healthcare professionals promptly identify abnormal gastrointestinal symptoms.

### 4.6 Standardized feeding protocol

The guidelines recommend that each NICU establish and implement a standardized feeding protocol or workflow (15). However, according to this survey, only 68.1% of the NICUs had established relevant documentation for VLBW infants. These protocols provide detailed and stringent specifications for enteral feeding formulas, volume targets, and HMF based on gestational age and birth weight (49), and could also incorporate personalized developmental intervention modules for preterm infants (50). The implementation of standardized feeding protocols not only meets nutritional requirements and accelerates the feeding process (51, 52) but also reduces the risk of complications such as central line-associated bloodstream infection (CLABSI) and phlebitis (51, 53), thereby improving developmental outcomes in VLBW infants (51). Therefore, NICUs across diverse geographic regions should develop standardized feeding protocols and routinely update them with current evidence.

### 4.7 Implications for practice and policy

All NICUs, including those in Shandong Province, should develop and regularly update standardized feeding protocols for VLBW infants that are consistent with Chinese and international guidelines, incorporating explicit criteria for early initiation and faster advancement of feeding in clinically stable infants. Once the infant is clinically stable, enteral feeding should be initiated as early as possible, avoiding delays due to non-evidence-based concerns or routine GRV checks. After reaching a predefined feeding volume threshold, routine HMF should be promoted. If HMF is unavailable or unaffordable, PTF may be used selectively as a fortifier. AF may offer greater individualization but requires more frequent monitoring and higher resource investment, so it should be reserved for settings with adequate staffing and lab capacity. Routine GRV monitoring should be reduced in stable infants, replaced by structured clinical feeding tolerance assessments or risk prediction models. Tools such as POCUS and NIRS may be considered only where the necessary equipment and expertise are available to support enteral feeding management. Finally, Shandong’s conservative advancement practices can safely align with evidence for faster feeding from comparable resource-limited settings, provided clinical stability is assured.

### 4.8 Limitations

The limitations of this study include selection bias and regional bias. Although 47 Level II and Level III NICUs participated in this study, its scope was restricted to a single province, introducing a degree of bias. The healthcare and economic standards of Shandong Province were often considered representative of both Eastern China and the nation. Still, these findings should be generalized to China’s overall level with caution, particularly given the significant differences in healthcare philosophies between the north and south. This study was based on self-reported survey data without validation against institutional practices and lacked patient-level clinical outcomes, increasing the risk of bias. In addition, this study is a descriptive survey and narrative comparison, which does not use a formal scoring system to analyze guideline adherence. Therefore, the conclusions of this study may be overstated and should be interpreted with caution. Future research should account for regional variations, levels of clinical practice, and the number of healthcare institutions. More categories of institutions, including general, maternity, and child specialized, and traditional Chinese medicine hospitals, as well as institutions with more detailed NICU grades, such as level III (a-c) and level II (a-c), should be invited to analyze the current situation of enteral feeding practice and the adherence to guidelines. Through more representative comparisons and analyses, a more suitable feeding strategy for VLBW infants in China could be developed, along with a clearer direction for future progress.

## 5 Conclusion

This multicentre study, which included NICUs across different levels and hospital categories, aimed to investigate the current status of enteral feeding for VLBW infants. It not only provided regional data but also analyzed implementation gaps between evidence and clinical practice. The research found that, although most clinical practices were aligned with evidence and guidelines, there are still discrepancies in certain specific procedural details. In the future, these results should be further generalized through broader investigations in order to support and advance the quality improvement of neonatal nutrition practice across other regions.

## Data Availability

The raw data supporting the conclusions of this article will be made available by the authors, without undue reservation.
